# Inferior Vena Cava Thrombi Related to Intravascular Temperature Management Catheters

**DOI:** 10.31662/jmaj.2024-0300

**Published:** 2025-06-13

**Authors:** Shuhei Tada, Yuki Kondo, Tomoya Okazaki

**Affiliations:** 1 Department of Emergency and Critical Care Medicine, Tokyo Bay Urayasu Ichikawa Medical Center, Urayasu, Japan

**Keywords:** intravascular temperature management catheter, inferior vena cava thrombosis, ultrasound sonography

A 90-year-old woman and a 44-year-old man were admitted after out-of-hospital cardiac arrests.

After the return of spontaneous circulation, they were unconscious. Targeted temperature management was implemented. In both patients, a heparin-coated intravascular temperature management (IVTM) catheter was placed through the femoral vein and was removed within 4 days.

Despite proper catheter use and vein thrombosis prophylaxis, hyperechoic, large, mobile, mural thrombi in the inferior vena cava (IVC) were detected on day 6 in the woman and day 8 in the man via B-mode ultrasound (Figure 1). Both patients were followed up while receiving continuous unfractionated heparin.

**Figure 1.**
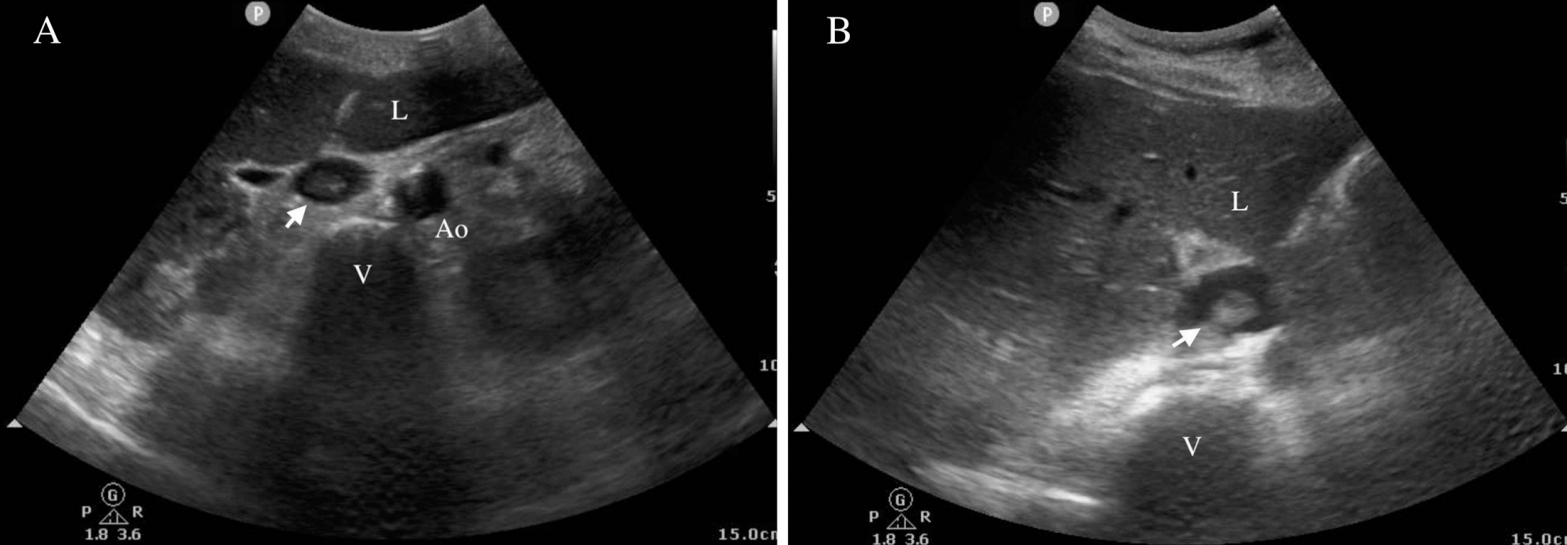
Sonographic image of the thrombi in the IVC. Thrombi in the IVC of patients 1 (A) and 2 (B). Both thrombi were hyperechoic, large, mobile, and mural (white arrows). Ao: aorta; IVC: inferior vena cava; L: liver; V: vertebral body.

Several studies reported IVTM catheter-related thrombus formation in the IVC ^(1), (2)^. Screening for IVC thrombosis typically includes duplex ultrasound, computed tomography, or magnetic resonance imaging ^(3)^. B-mode ultrasound is easier to use than other modalities in screening IVC thrombosis. These cases highlight the value of ultrasound for early detection of IVC thrombosis following IVTM catheter use.

## Article Information

### Conflicts of Interest

None

### Author Contributions

Shuhei Tada contributed to patient care, planning, conduct, and writing up the work. Yuki Kondo and Tomoya Okazaki provided critical revision of the report. All authors reviewed and approved the final version.

### Patient Consent

Consent to publish the details of the present case was obtained from the patient.
